# Post-translational modifications of beta-amyloid alter its transport in the blood-brain barrier *in vitro* model

**DOI:** 10.3389/fnmol.2024.1362581

**Published:** 2024-03-07

**Authors:** Kseniya B. Varshavskaya, Irina Yu Petrushanko, Vladimir A. Mitkevich, Evgeny P. Barykin, Alexander A. Makarov

**Affiliations:** Engelhardt Institute of Molecular Biology, Moscow, Russia

**Keywords:** Alzheimer's disease, blood-brain barrier, beta-amyloid, post-translational modifications, rage, caveolin-dependent endocytosis, clathrin-dependent endocytosis

## Abstract

One of the hallmarks of Alzheimer's disease (AD) is the accumulation of beta-amyloid peptide (Aβ) leading to formation of soluble neurotoxic Aβ oligomers and insoluble amyloid plaques in various parts of the brain. Aβ undergoes post-translational modifications that alter its pathogenic properties. Aβ is produced not only in brain, but also in the peripheral tissues. Such Aβ, including its post-translationally modified forms, can enter the brain from circulation by binding to RAGE and contribute to the pathology of AD. However, the transport of modified forms of Aβ across the blood–brain barrier (BBB) has not been investigated. Here, we used a transwell BBB model as a controlled environment for permeability studies. We found that Aβ_42_ containing isomerized Asp7 residue (iso-Aβ_42_) and Aβ_42_ containing phosphorylated Ser8 residue (pS8-Aβ_42_) crossed the BBB better than unmodified Aβ_42_, which correlated with different contribution of endocytosis mechanisms to the transport of these isoforms. Using microscale thermophoresis, we observed that RAGE binds to iso-Aβ_42_ an order of magnitude weaker than to Aβ_42_. Thus, post-translational modifications of Aβ increase the rate of its transport across the BBB and modify the mechanisms of the transport, which may be important for AD pathology and treatment.

## 1 Introduction

Alzheimer's disease (AD) is the most common neurodegenerative disease, accounting for 60%−80% of all cases of dementia (Sonkusare et al., 2005). AD is characterized by various pathological markers in the brain, such as the accumulation of beta-amyloid peptide (Aβ), which can form senile plaques, intracellular accumulation of neurofibrillary tangles formed by hyperphosphorylated tau protein, and progressive loss of nerve cells (Scheltens et al., 2016). Most cases of AD are sporadic and aging is considered a major risk factor for AD, but the pathways through which aging triggers the development of the disease are still unclear. It has been suggested that aging may induce post-translational modifications of Aβ (PTMs), which enhance its pathogenic properties (Moro et al., 2018). Thus, Aβ is capable of undergoing various PTMs that are triggered by enzymes or low molecular weight substances, as well as spontaneously (Barykin et al., 2017). Some of these modifications are isomerization of the aspartic acid residue at position 7 (iso-Aβ) and phosphorylation at serine 8 (pS8-Aβ). These modifications are located in the metal-binding domain of Aβ, which regulates its zinc-dependent oligomerization (Zirah et al., 2006; Barykin et al., 2018) and interaction with receptors (Barykin et al., 2018; Forest et al., 2018). In amyloid plaques, iso-Aβ was found to constitute more than 50% of all Aβ molecules (Mukherjee et al., 2021). Iso-Aβ has an increased ability to oligomerize (Shimizu et al., 2005), is more toxic (Mitkevich et al., 2013) and demonstrates resistance to proteolysis (Kummer and Heneka, 2014). At the same time, the level of iso-Aβ increases with age and in patients with AD (Moro et al., 2018). PS8-Aβ was detected in brain tissue of both patients with AD and AD model mice. It is localized both in amyloid plaques and in the cytoplasm of neurons, and compared to unmodified Aβ has increased neurotoxicity *in vitro* (Jamasbi et al., 2017) and higher resistance to degradation by an insulin-degrading enzyme (Kummer and Heneka, 2014). Thus, pS8-Aβ and iso-Aβ are important isoforms that differ significantly in properties from intact Aβ. The changes in the homeostasis of these isoforms may trigger pathological events contributing to development of AD.

Numerous studies have shown that AD is accompanied by a disruption of the blood-brain barrier (BBB), which occurs at an early stage of the disease (Nation et al., 2019; Barisano et al., 2022). The BBB controls the entry of Aβ from plasma into the brain via the RAGE receptor, as well as the clearance of Aβ from the brain into the peripheral circulation via the LRP-1 receptor (Zenaro et al., 2017). Disruption of these BBB functions can lead to pathological accumulation of Aβ in the brain and manifestation of AD symptoms. Increasing evidence indicates that Aβ from blood can enter the brain and serve as a trigger for the disease (Bu et al., 2018; Sun et al., 2021). Interestingly, peripheral injection of synthetic Aβ_42_ into the bloodstream did not lead to the formation of amyloid plaques in the brains of mouse models of AD. However, intravenous injections of modified forms of Aβ altered the pathology of AD: the injection of iso-Aβ accelerated the amyloidogenesis (Kozin et al., 2013), while injection of pS8-Aβ reduced the number of amyloid plaques in the brain of transgenic mice (Barykin et al., 2018). This evidence suggests that pathogenic isoforms of Aβ may arise in the circulatory system, after which they penetrate the brain and contribute to AD pathology (Kozin and Makarov, 2019). However, the transport of modified forms of Aβ across the BBB has not been previously studied.

In this work, we compared the efficiency of transport of Aβ_42_, pS8-Aβ_42_, and iso-Aβ_42_ through a monolayer of BBB endothelial cells, and also established the contribution of clathrin- and caveolin-dependent mechanisms to this process. It was also determined how modifications of Aβ affect its affinity for RAGE.

## 2 Materials and methods

### 2.1 Preparation of synthetic beta-amyloid peptides

Synthetic beta-amyloid peptides: Aβ_42_ and iso-Aβ_42_ were obtained from Lifetein (Somerset, NJ, USA). PS8-Aβ_42_ and Aβ_1 − 16_ were obtained from Biopeptide (San Diego, CA, USA). Aβ_17 − 42_ was obtained from Verta (Saint-Petersburg, Russia). The amino acid sequence of the peptides is shown in Table 1.

**Table 1:** Amino acid sequence of Aβ and its isoforms.

**Peptide**	**Sequence**
Aβ_42_	[H2N]-DAEFRHDSGYEVHHQKLVFFAEDVGSNKGAIIGLMVGGVVIA-[COOH]
iso-Aβ_42_	[H2N]-DAEFRH[isoD]SGYEVHHQKLVFFAEDVGSNKGAIIGLMVGGVVIA-[COOH]
pS8-Aβ_42_	[H2N]-DAEFRHD[pS]GYEVHHQKLVFFAEDVGSNKGAIIGLMVGGVVIA-[COOH]
Aβ_1 − 16_	[H2N]-DAEFRHDSGYEVHHQK-[COOH]
Aβ_17 − 42_	[H2N]-LVFFAEDVGSNKGAIIGLMVGGVVIA-[COOH]

Peptides were monomerized using hexafluoroisopropanol (Fluka), aliquoted and dried as described in Barykin et al. (2018). An aliquot of Aβ was dissolved in 10 μl of dimethyl sulfoxide (DMSO) (Sigma-Aldrich, St. Loius, MO, USA) at room temperature for an hour to obtain a 1.25 mM stock solution and then diluted to 1 μM using serum-free DMEM media for Aβ transport experiments.

### 2.2 Cell culture

Mouse brain endothelial cell line bEnd.3, obtained from the American Type Culture Collection, was cultured at 37°C in an atmosphere of 5% CO_2_ in Dulbecco's Modified Eagles Medium (DMEM; Gibco, ThermoFisher Scientific, Waltham, MA, USA) containing 4.5 g/l glucose, 1% GlutaMax (Gibco, ThermoFisher Scientific, Waltham, MA, USA), 100 units/mL penicillin, 100 μg/mL streptomycin (Sigma, St. Louis, MO, USA) with the addition of 10% fetal bovine serum (FBS; Gibco, USA).

### 2.3 *In vitro* model of the BBB

#### 2.3.1 Cell cultivation on transwell membrane

To simulate the BBB, a mono-cultured model based on bEnd.3 cells was used. BEnd.3 cells were cultured in transwell inserts (Greiner Bio-One, pore diameter 0.4 μm) submerged in the wells of a 12-well plate (Greiner Bio-One). BEnd.3 cells were seeded on the upper surface of the transwell membrane in an amount of 70 thousand per well and cultured for 7 days until confluence. The volume of DMEM medium (10% FBS) in the upper (luminal) compartment was 750 μl, in the lower (abluminal) compartment – 1 ml. Cell counting before seeding was carried out in a Goryaev chamber with preliminary staining of cells with damaged membranes with trypan blue (Invitrogen).

#### 2.3.2 Measuring the passage of Aβ isoforms through a monolayer of bEnd.3 cells

The transport of Aβ and its isoforms was studied in the transwell model. Aβ is able to bind albumin and other serum proteins (Biere et al., 1996). Therefore, before the experiment, the upper and lower sections of the transwell were washed with 500 and 1000 μL of serum-free DMEM, respectively. To study the transfer of Aβ from the luminal to abluminal compartment (modeling transport from the blood to the brain), the upper part of the transwell was filled with 300 μl of DMEM containing 1 μM Aβ. An appropriate amount of DMSO (0.08% DMSO) was added to control samples. The lower compartment was filled with 750 μl of DMEM. After adding the peptide, samples were taken from the lower part of the transwell in a volume of 200 μl after 2, 6, and 24 h. Each time after sampling, 200 μl of DMEM medium was added to the lower compartment. After 6 h of incubation, FBS was added to the upper compartment of each well to a final concentration of 5%. The concentration of Aβ in the samples was determined by enzyme-linked immunosorbent assay (ELISA). To account for dilution due to sampling and addition of DMEM medium, the concentration of Aβ was corrected using the following equation:


$$\begin{array}{l} C_{t}^{\prime }=C_{t}+\left(\frac{V}{V_{total}}\;\times \;C_{t-\;1}f\right) \end{array}$$

where ${\text{C}}_{\text{t}}^{\prime }$ is the concentration of Aβ at time t, taking into account dilution; C_t_ is the concentration of Aβ measured with ELISA at time t; C_t − 1_ - concentration of Aβ measured at the previous time point; V is the volume added to the lower compartment after sampling; V_total_ is the total volume in the lower compartment.

The permeability coefficients obtained by incubating cells with 1 μM and 100 nM Aβ are presented in the Supplementary Figure 1.

After each experiment, BBB permeability was assessed as described in section 2.3.3 to ensure that the cell monolayer was not disrupted by incubation with Aβ.

#### 2.3.3 Endothelial permeability measurement

To assess paracellular permeability of the endothelium, the fluorescent label sodium fluorescein (Sigma-Aldrich) was used. The lower and upper compartments of the transwell were washed with PBS (Gibco) and filled with HBSS buffer (Gibco): 250 and 750 μl in the upper and lower compartments, respectively. Then, 50 μl of 60 μg/ml sodium fluorescein dissolved in HBSS was added to the upper compartment to a final concentration of 10 μg/ml. Samples (100 μl) were taken from the lower compartment at 0, 15, 30, 45, and 60 min after the addition of the fluorescent label. Each time after sampling, 100 μl of HBSS was added to the lower compartment. The fluorescence intensity in the samples was measured on a SPARK plate reader (Tecan, Switzerland) with an excitation wavelength of 485 nm and a fluorescence recording wavelength of 535 nm. Dilution of sodium fluorescein at each sampling step was taking into account using the following equation:


$$\begin{array}{l} I_{t}^{\prime }=I_{t}+\left(\frac{V}{V_{total}}\;\times \;I_{t-\;1}\right) \end{array}$$

where ${\text{I}}_{\text{t}}^{\prime }$ is the fluorescence intensity at time t after dilution correction; I_t_ is the fluorescence intensity measured at time t; I_t − 1_ is fluorescence intensity measured at the previous time point; V is the volume added to the lower compartment after sampling; V_total_ is the total volume in the lower compartment.

#### 2.3.4 Study of the transport mechanisms of beta-amyloid and its isoforms

Various inhibitors were used to study the transport mechanisms of Aβ and its isoforms. The contribution of RAGE to the transport of Aβ across the endothelial monolayer was assessed using the antagonist of this receptor FPS-ZM1 (Sigma) at a concentration of 20 μM (to obtain a stock solution, FPS-ZM1 was dissolved in DMSO to a concentration of 305 mM). To study the caveolin-dependent transport of Aβ isoforms, the inhibitor filipin (Sigma) was used at a concentration of 3 μg/ml (to obtain a stock solution, filipin was dissolved in DMSO to a concentration of 5 mg/ml). An equivalent amount of DMSO was added to the control samples. The contribution of clathrin-dependent endocytosis was assessed using chlorpromazine (Merck) at a concentration of 5 μg/ml (to obtain a stock solution, chlorpromazine was dissolved in DMEM). These concentrations were selected based on literature data and tested for toxicity to bEnd.3 cells using MTT (Filipin) and WST (FPS-ZM1 and chlorpromazine) assays according to the manufacturer's protocol (Supplementary Figure 2). Before experiments, cells were preincubated with inhibitors added to the upper transwell compartment for 1 h, after which they were filled with solutions containing the inhibitor and 1 μM Aβ, and samples were taken from the lower compartment after 2, 6 and 24 h.

### 2.4 ELISA

The concentration of Aβ and its isoforms was measured using sandwich ELISA. BAM113cc antibodies (HyTest), which recognize the C-terminus of Aβ, were added to a 96-well ELISA plate (NEST) in a volume of 100 μl (0.5 ng/μl) and incubated overnight at +4°C. The wells of the plate were washed 2 times with 200 μl of PBST (0.05% Tween20) and blocked in 1% BSA (Dia-m) in PBST at room temperature and shaking for 3–4 h. Then the plate was washed 2 times with 200 μl of PBST. BAM7cc antibodies (HyTest) conjugated to HRP were added (50 μl per well, 2 ng/μl), then standards and experimental samples were added (50 μl per well). Samples were incubated overnight at +4°C, washed 6 times with 200 μl PBST and analyzed using TMB (Merck). Absorbance was measured at 450 nm using a Multiskan FC Microplate Photometer (Thermo Fisher Scientific). The calibration curves are presented in the Supplementary Figure 3).

### 2.5 Determination of parameters of interaction of Aβ and its isoforms with RAGE

The His-tag-containing sRAGE protein (Abcam) was stained with a fluorescent dye using the second-generation Monolith His-Tag Labeling Kit RED-tris-NTA according to the manufacturer's protocol. Aliquots of Aβ_42_, pS8-Aβ_42_, iso-Aβ_42_, and Aβ_17 − 42_ were dissolved in DMSO to a concentration of 5 mM, after which a series of dilutions were prepared to obtain solutions with Aβ concentrations from 6.1 nM to 200 μM. Aliquots of Aβ_1 − 16_ were dissolved in PBS to a concentration of 2.5 mM, after which a series of dilutions were prepared (the final concentration of Aβ_1 − 16_ in the samples varied from 38.1 nM to 1.25 mM). Samples were loaded into Monolith NT.115 Premium capillaries. The concentration of RED-tris-NTA-labeled sRAGE was constant (50 nM). All samples contained 4% DMSO and 20% glycerol. Microthermophoresis was performed using a Monolith NT.115 system (Nano Temper Technologies GmbH). Data analysis was performed using MO. Affinity Analysis v.2.3 software (Nano Temper Technologies GmbH).

### 2.6 Measurement of intracellular concentrations of Aβ and its isoforms

BEnd.3 cells were seeded into a 12-well plate (Greiner Bio-One) at 35 thousand per well and cultured for a week in DMEM (10% FBS), after which they were incubated with 1 μM Aβ_42_, pS8-Aβ_42_ or iso-Aβ_42_ within 24 h in serum-free DMEM. Cells were washed three times with PBS, frozen in liquid nitrogen, and stored at −80°C overnight. Cells were lyzed on ice for 15 min with 250 μl of IP Lysis Buffer (Pierce) containing protease and phosphatase inhibitors (Roche) per well. The cells were removed using a scraper and placed in tubes, after which the cells were lysed for 1 h at +4°C with shaking. The cell lysate was centrifuged for 10 min at 16000 g, +4°C, and the supernatant was collected. The amount of protein in the lysates was determined using a BCA assay kit (Sigma) according to the manufacturer's protocol. The concentration of Aβ and its isoforms in cell lysates was measured using ELISA as described above.

### 2.7 Statistical data processing

Experimental data are presented as the mean of independent experiments ± standard deviations (SD) or as a boxplot showing the median, lower and upper quartiles, minimum and maximum values of the sample. The number of independent experiments is indicated in the figure legends. The normality of the distribution was checked using the Kolmogorov-Smirnov test, and outliers were analyzed using the Q-test. Statistical differences between experimental groups for normally distributed samples were determined using Student's t test (when comparing two groups) or One-way ANOVA (when comparing multiple groups) using Tukey's test for multiple comparisons. Differences were considered statistically significant at p < 0.05. Statistical analysis was performed using GraphPad Prism 8.0.2 software.

## 3 Results

### 3.1 Isomerized and phosphorylated Aβ pass through the BBB model more efficiently than unmodified Aβ

**Figure 1:**
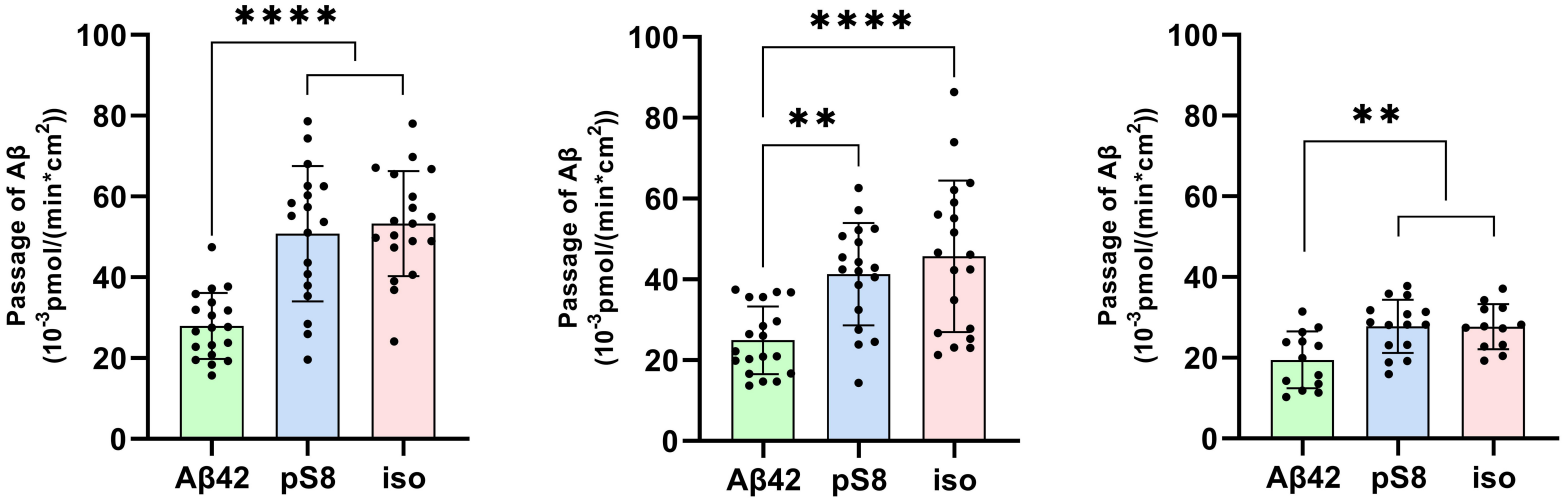
Passage of 1 μM Aβ_42_, pS8-Aβ_42_ and iso-Aβ_42_ through a monolayer of bEnd.3 cells from the upper transwell compartment to the lower compartment at 2, 6 and 24 hours. The amounts (pmol) of Aβ_42_, pS8-Aβ_42_ and iso-Aβ_42_ in the lower compartment measured by sandwich ELISA normalized by incubation time (min) and transwell area (cm^2^) are presented. Number of values in each group *n* = 15–19 representing 6 independent experiments. ^**^p < 0.01, ^****^p < 0.0001.

The passage of Aβ_42_, pS8-Aβ_42_ and iso-Aβ_42_ across the BBB was measured in a transwell system. It was found that pS8-Aβ_42_ and iso-Aβ_42_ are transported by endothelial cells from the luminal (upper) transwell compartment to the abluminal (lower) compartment more efficiently than Aβ_42_ (Figure 1). Thus, the transport efficiency of pS8-Aβ_42_ was 1.8, 1.7 and 1.4 times higher than that of the unmodified peptide after 2, 6 and 24 h of incubation, respectively. Transport of iso-Aβ_42_ through the endothelium was 1.9, 1.8 times (at 2 and 6 h, respectively) and 1.4 times (at 24 h) more efficient than Aβ_42_ transport. It can be seen that the transport rate is lower after 24 h of incubation compared to 2 and 6 h. A possible reason for this is that prolonged incubation can lead to degradation or aggregation of Aβ.

**Figure 2:**
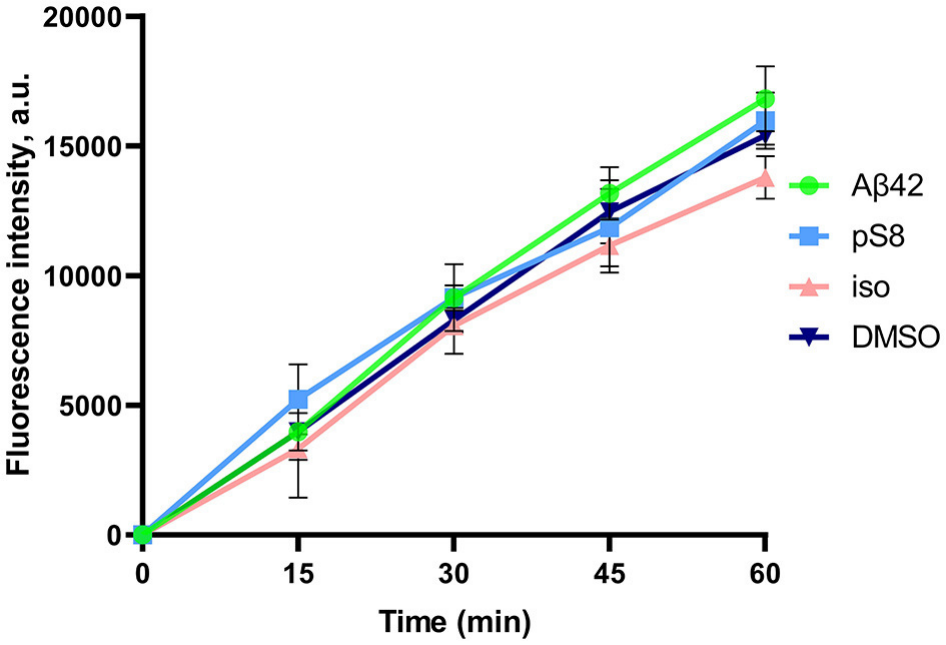
Efficiency of sodium fluorescein passage through a monolayer of bEnd.3 cells. Prior to the measurement, the cells were incubated with 0.08% DMSO, or with 1 μM Aβ_42_, pS8-Aβ_42_, or iso-Aβ_42_ for 24 h. Fluorescence intensity values in the lower transwell compartment are shown. Number of independent replicates *n* = 3.

After the experiment, the integrity of the endothelium was checked using sodium fluorescein (Figure 2). The permeability of cell monolayer to sodium fluorescein did not differ between control cells and cells treated with amyloid peptides. This indicates that incubation with amyloid peptides did not affect the integrity of the bEnd.3 cell monolayer.

### 3.2 The mechanism of transport of Aβ_42_, pS8-Aβ_42_ and iso-Aβ_42_ across the BBB is different

**Figure 3:**
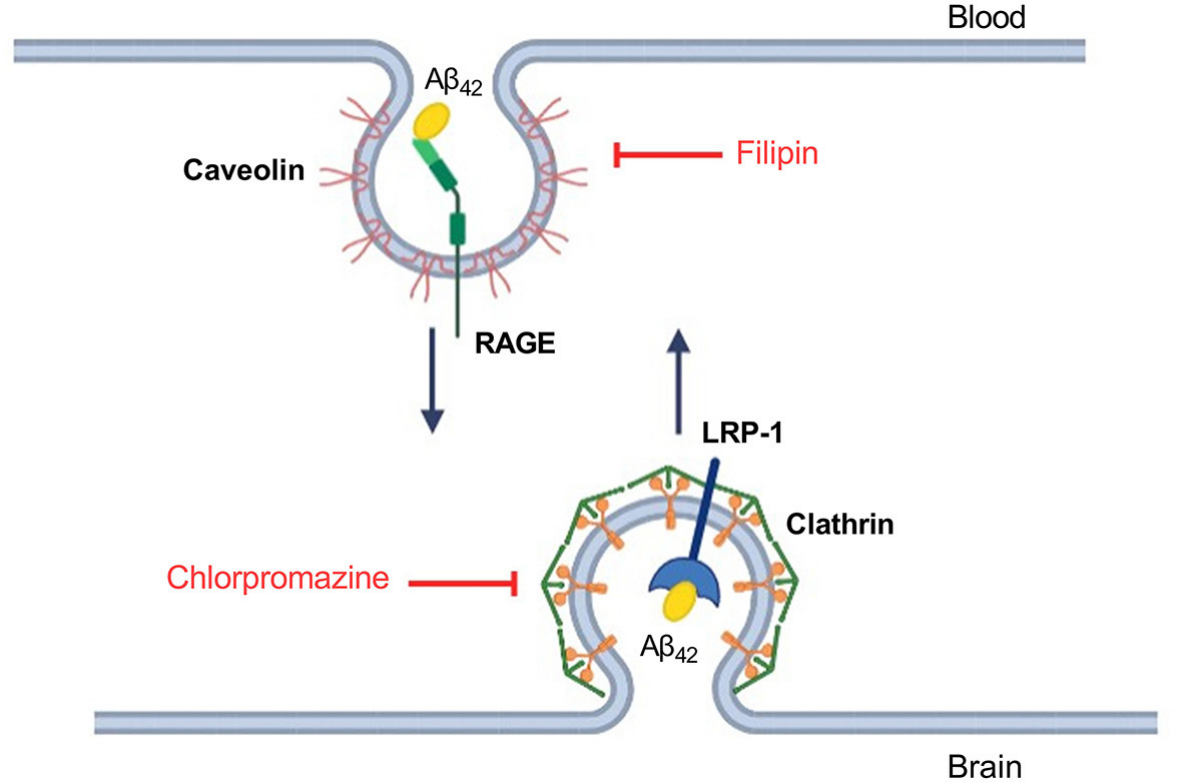
Schematic representation of Aβ_42_ transport through the endothelium of the BBB and the underlying molecular mechanisms. Inhibitors affecting caveolin- and clathrin-dependent endocytosis are indicated in red. Filipin binds cholesterol in the membrane and interferes with caveolae formation (Abulrob et al., 2005). Chlorpromazine affects the complex of adapter proteins AP-2 involved in clathrin-dependent endocytosis (Daniel et al., 2015).

The different efficiency of passage of Aβ isoforms through the BBB model may indicate differences in the mechanisms of their transcellular transport. It is known that Aβ_42_ enters the brain from the blood through the mechanism of caveolin-dependent endocytosis, binding to RAGE, and Aβ_42_ is cleared from the brain to the blood mainly through the LRP-1 receptor via clathrin-dependent endocytosis (Zhu et al., 2018) (Figure 3). In order to study the contribution of various mechanisms to the transport of Aβ and its isoforms, an inhibitor of caveolin-dependent endocytosis, filipin, and an inhibitor of clathrin-dependent endocytosis, chlorpromazine, were used.

**Figure 4:**
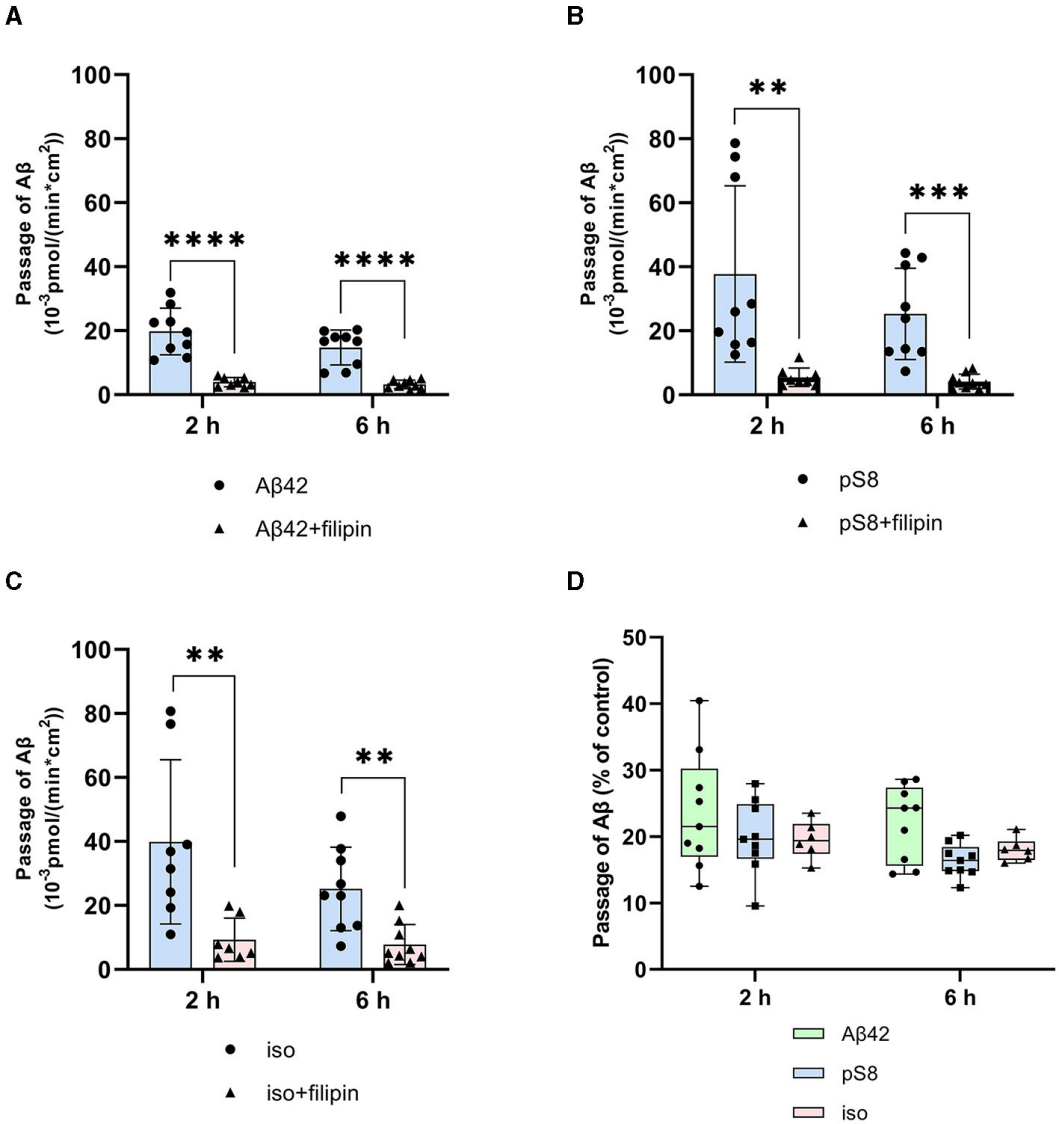
Effects of filipin on the efficiency of Aβ_42_ **(A)**, pS8-Aβ_42_ **(B)** and iso-Aβ_42_ **(C)** transport through a monolayer of bEnd.3 cells in the transwell model. The amounts (pmol) of Aβ_42_, pS8-Aβ_42_ and iso-Aβ_42_ in the lower compartment normalized by incubation time (min) and transwell area (cm^2^) after 2 and 6 hours of incubation with amyloid peptides in the absence or presence of filipin are shown. **(D)** Comparison of the degree of inhibition of filipin Aβ_42_, pS8-Aβ_42_ and iso-Aβ_42_, where transport of the peptides in the absence of the inhibitor was taken as 100% (not shown). Summarized data from three independent experiments are presented, the number of values in each group *n* = 6–9, **p < 0.01, ***p < 0.001, ****p < 0.0001.

It was found that filipin inhibits not only the transport of Aβ_42_, as reported previously (Zhu et al., 2018), but also the passage of pS8-Aβ_42_ and iso-Aβ_42_ (Figures 4A–C). The degree of inhibition for all of the isoforms was about 75% (Figure 4D).

**Figure 5:**
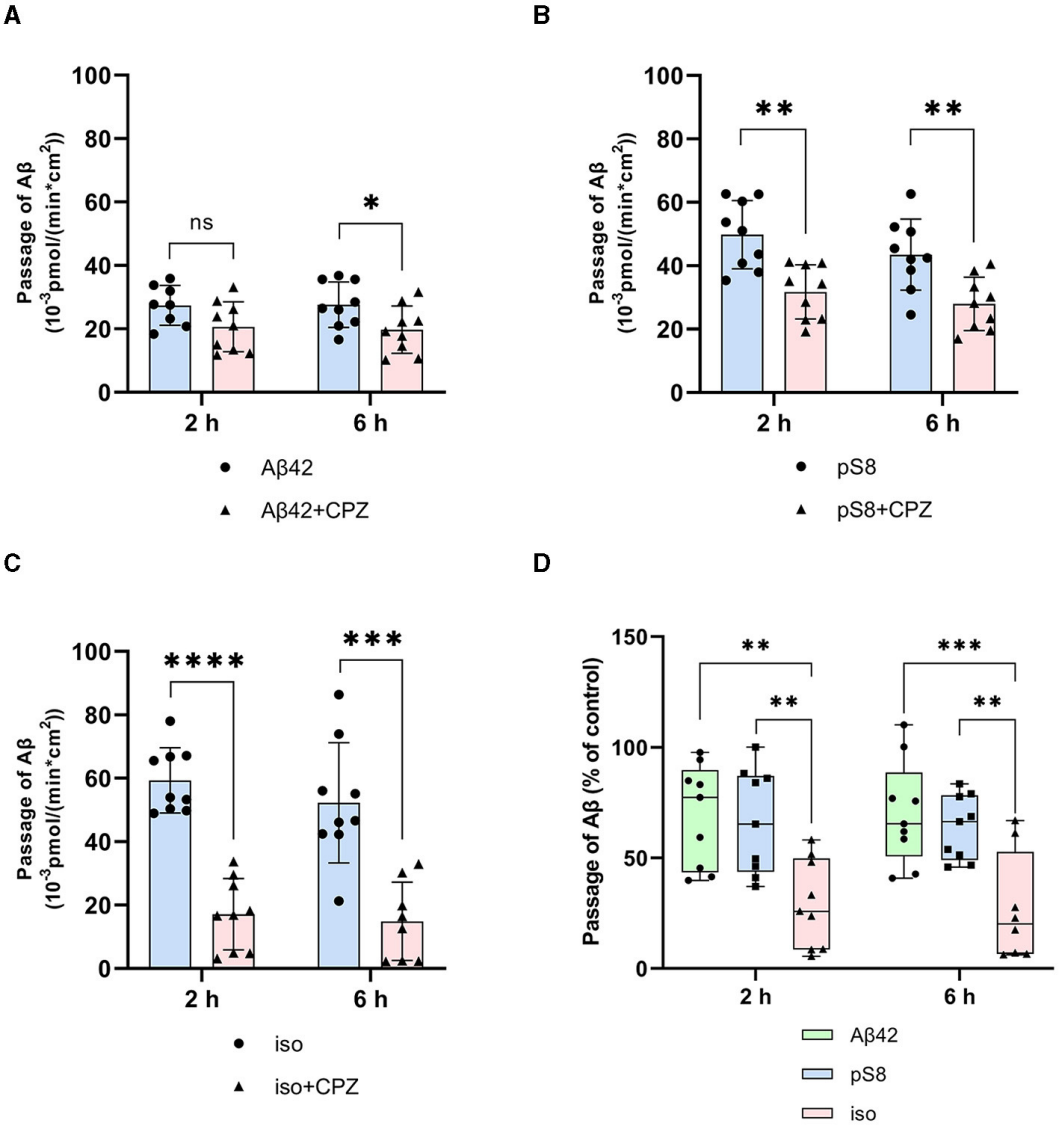
Effects chlorpromazine (CPZ) on the efficiency of Aβ_42_ **(A)**, pS8-Aβ_42_ **(B)** and iso-Aβ_42_ **(C)** transport through a monolayer of bEnd.3 cells in the transwell model. The amounts (pmol) of Aβ_42_, pS8-Aβ_42_ and iso-Aβ_42_ in the lower compartment normalized by incubation time (min) and transwell area (cm^2^) after 2 and 6 h of incubation with amyloid peptides in the absence or presence of CPZ are shown. **(D)** Comparison of the degree of inhibition of CPZ Aβ_42_, pS8-Aβ_42_ and iso-Aβ_42_, where transport of the peptides in the absence of the inhibitor was taken as 100% (not shown). Summarized data from three independent experiments are presented, the number of values in each group *n* = 6–9, ns - not significant, *p < 0.05, **p < 0.01, ***p < 0.001, ****p < 0.0001.

Chlorpromazine inhibited the transport of Aβ_42_ and pS8-Aβ_42_ by 30% (Figures 5A, B). Surprisingly, the efficiency of iso-Aβ_42_ passage through the endothelium in the presence of chlorpromazine decreased by about 75% (Figure 5C). The degree of inhibition for iso-Aβ_42_ was different from other isoforms (Figure 5D). This indicates a difference in the contribution of clathrin and caveolin-dependent endocytosis to the transfer of Aβ isoforms across the endothelium in BBB model.

### 3.3 Aβ modifications affect the interaction with RAGE

**Figure 6:**
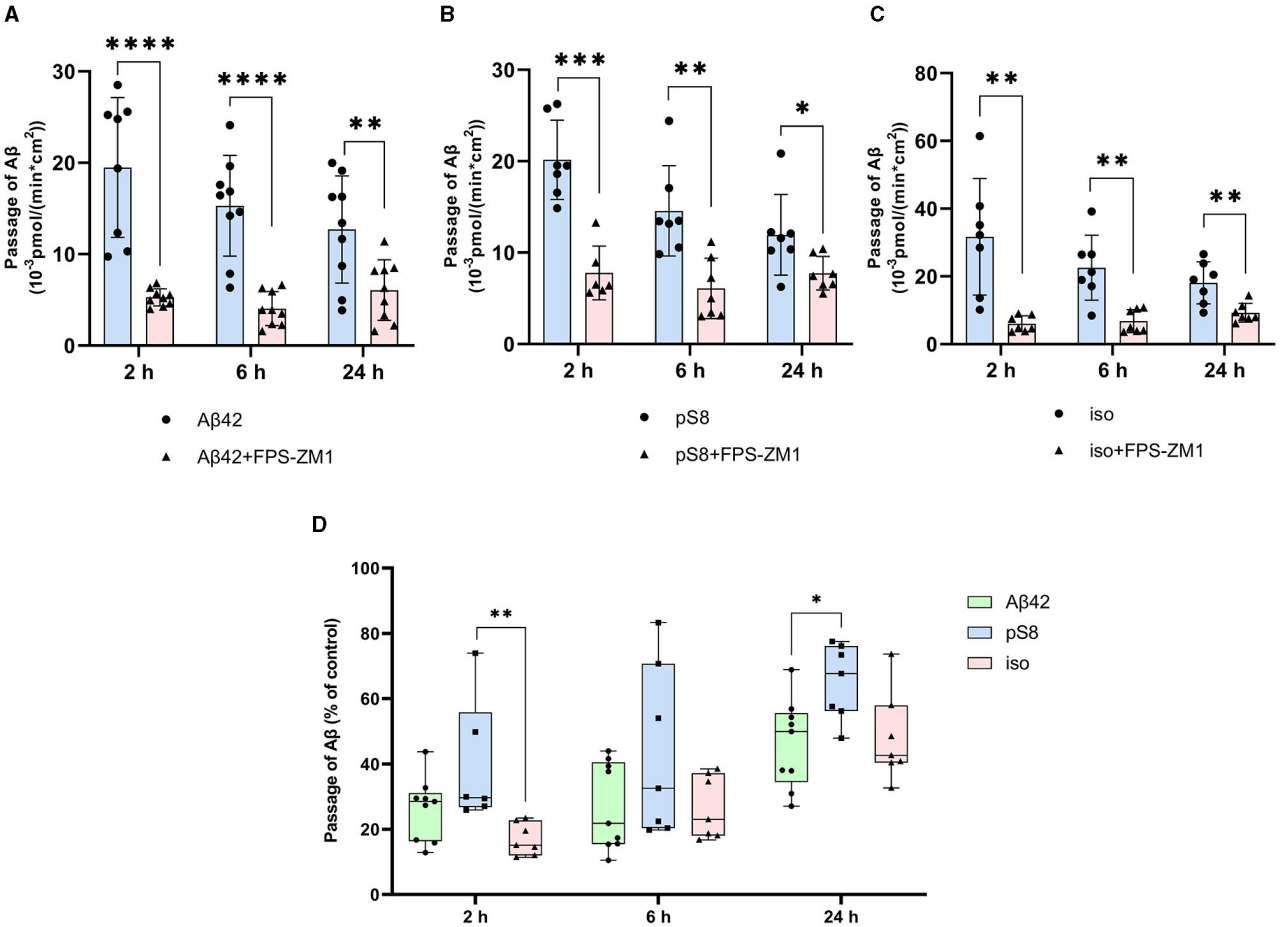
Effect of the RAGE antagonist FPS-ZM1 on the efficiency of transport of Aβ_42_ **(A)**, pS8-Aβ_42_ **(B)** and iso-Aβ_42_ **(C)** through a monolayer of bEnd.3 cells in the transwell model. The amounts (pmol) of Aβ_42_, pS8-Aβ_42_ and iso-Aβ_42_ in the lower compartment normalized by incubation time (min) and transwell area (cm^2^) after 2, 6 and 24 hours of incubation with amyloid peptides in the absence or presence of FPS-ZM1 are shown. **(D)** Comparison of the degree of inhibition of FPS-ZM1 Aβ_42_, pS8-Aβ_42_ and iso-Aβ_42_, where transport of the peptides in the absence of the inhibitor was taken as 100% (not shown). Summarized data from three independent experiments are presented, the number of values in each group *n* = 6–9, *p < 0.05, **p < 0.01, ***p < 0.001, ****p < 0.0001.

Differences in the efficiency of passage of Aβ_42_, pS8-Aβ_42_, and iso-Aβ_42_ through the BBB endothelium may be a result of the differences in interaction with RAGE. To study RAGE/Aβ interaction in our BBB model, inhibitor FPS-ZM1, which blocks the binding of Aβ_42_ to the V domain of the receptor (Deane et al., 2012), was used. FPS-ZM1 was found to significantly reduce the efficiency of transport of all Aβ isoforms through a monolayer of bEnd.3 cells (Figure 6). However, FPS-ZM1 inhibited the passage of Aβ_42_ and iso-Aβ_42_ more efficiently than that of pS8-Aβ_42_ for 2 and 24 h of incubation (Figure 6D).

**Figure 7:**
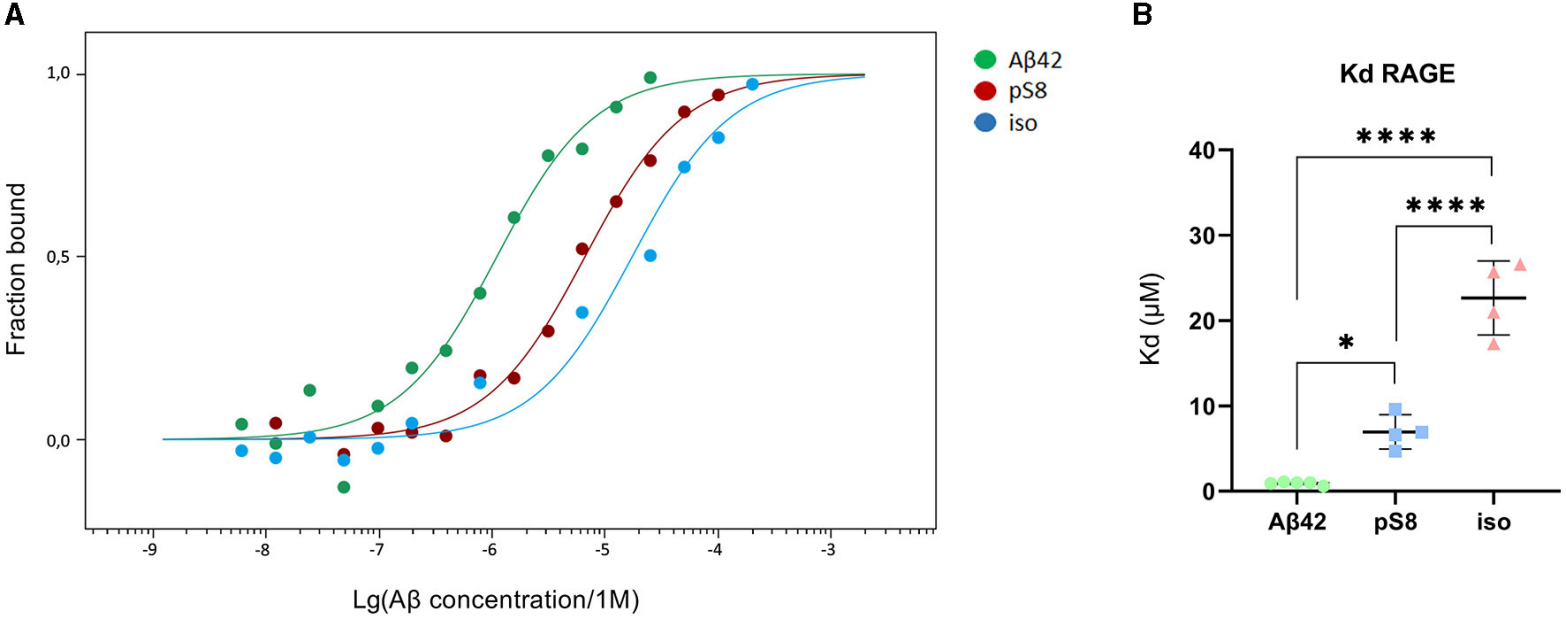
Interaction of Aβ isoforms with sRAGE. **(A)** MST curves showing the fraction of RAGE which is in the complex with the peptide at different concentrations of Aβ and its isoforms. **(B)** Values of dissociation constants (Kd) for complexes of Aβ isoforms with sRAGE. Number of replicates in each group *n* = 4–5, *p < 0.05, ****p < 0.0001.

**Figure 8:**
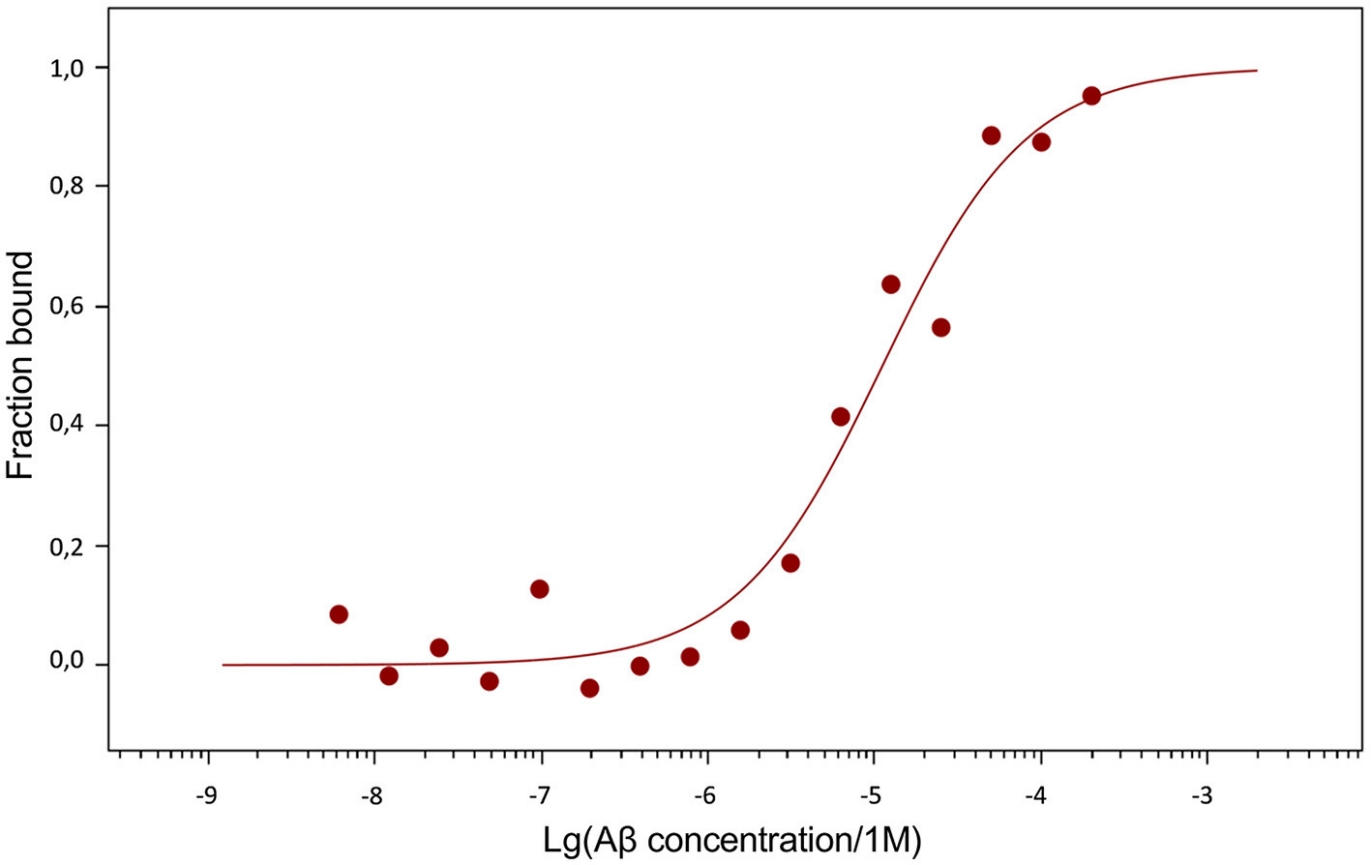
MST curve illustrating the interaction of Aβ_17 − 42_ with sRAGE.

The dissociation constants (Kd) of Aβ_42_, pS8-Aβ_42_ and iso-Aβ_42_ with the soluble extracellular part of RAGE (sRAGE), determined using microscale thermophoresis (MST), were 1.0 ± 0.2 μM, 7 ± 2 μM and 23 ± 4 μM, respectively (Figure 7). In addition, it was found that the C-terminal domain of Aβ_17 − 42_ forms a complex with sRAGE with Kd = 10 ± 5 μM (Figure 8). The interaction of sRAGE with the N-terminal domain of Aβ_1 − 16_ was not detected. Thus, C-terminal domain is the main factor in the interaction of amyloid peptides with sRAGE, and the N-terminal domain modulates this interaction.

### 3.4 Aβ_42_, pS8-Aβ_42_ and iso-Aβ_42_ accumulate differently in bEnd.3 cells

**Figure 9:**
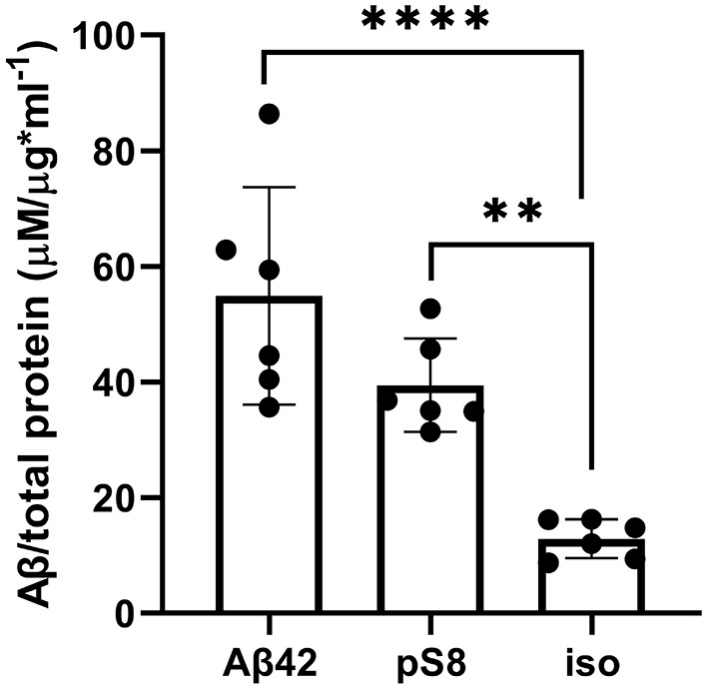
Levels of Aβ_42_, pS8-Aβ_42_ and iso-Aβ_42_ in bEnd.3 cells after 24 h of incubation with these peptides (1 μM). The data were obtained on cell lysates using ELISA. The concentrations of peptides normalized to the total protein (μg) in the samples are presented. Number of replicates in each group *n* = 6, **p < 0.01, ****p < 0.0001.

The reduced affinity of pS8-Aβ_42_ and iso-Aβ_42_ for RAGE compared to Aβ_42_ may cause lesser accumulation of these peptides inside cells and a more efficient transport to the abluminal side. To test this hypothesis, the intracellular levels of Aβ_42_, pS8-Aβ_42_, and iso-Aβ_42_ were measured after incubating cells with 1 μM of these peptides for 24 h (Figure 9). It was shown that intact Aβ_42_ accumulates better inside cells, while the level of accumulation of iso-Aβ_42_ is minimal compared to other peptides.

## 4 Discussion

It is known that Aβ is expressed not only in the brain, but also in cells of other organs and tissues: kidneys and adrenal glands, heart, liver, spleen, pancreas, as well as in muscles, blood cells and endothelium (Roher et al., 2009). Significant amounts of Aβ have been found in human red blood cells, and the Aβ_42_/Aβ_40_ ratio in red blood cells is higher than in plasma (Kiko et al., 2012). Platelets also express large amounts of amyloid precursor protein (APP) and release beta-amyloid. Once activated, for example by bleeding, platelets can secrete significant amounts of Aβ into the blood (Humpel, 2017; Carbone et al., 2021). Several studies have shown that platelets from people with AD may have a greater tendency to become activated and therefore release Aβ into the blood (Carbone et al., 2021).

Increasing evidence indicates that peripheral Aβ can penetrate into the brain and play a significant role in the pathogenesis of AD. Thus, peripheral inoculation of brain extracts containing Aβ led to amyloidosis in the brain of mice (Eisele et al., 2010, 2014; Burwinkel et al., 2018). It has also been shown that increasing the concentration of peripheral Aβ significantly reduces its removal from the brain (Marques et al., 2009). Inhibition of RAGE-ligand interaction suppressed brain Aβ accumulation in a transgenic mouse model (Deane et al., 2003). The important role of peripheral Aβ and its ability to enter the brain and trigger AD pathology was further highlighted in a parabiosis model in which the circulatory systems of a transgenic mouse with AD-like pathology and a wild-type mouse were connected. Using this model, the researchers demonstrated that human Aβ derived from a transgenic animal entered the wild-type mouse brain and initiated AD-like pathology, including tau hyperphosphorylation, neurodegeneration, neuroinflammation, impaired hippocampal long-term potentiation, and amyloid plaque formation (Bu et al., 2018). Another study demonstrated the contribution of Aβ produced by blood cells to the pathogenesis of AD: when bone marrow was transplanted from transgenic mice to wild-type mice, the latter showed signs of AD pathology (Sun et al., 2021). A number of data indicate that induction of AD requires not just an increase in the concentration of Aβ_42_, but the appearance of pathogenic forms carrying post-translational modifications (Kummer and Heneka, 2014; Barykin et al., 2017). Thus, intravenous administration of iso-Aβ_42_ accelerates amyloidogenesis in the brain of transgenic mice modeling AD (Kozin et al., 2013), and introduction of pS8-Aβ_42_ into the blood, on the contrary, reduces the number of amyloid plaques (Barykin et al., 2018). At the same time, intravenous administration of the unmodified peptide does not affect the formation of amyloid plaques in the brain of model mice. It is possible that modified forms of Aβ arise in the circulatory system, after which they enter the brain and contribute to AD pathology (Kozin and Makarov, 2019).

In this work, we compared the efficiency of transport of Aβ isoforms in an *in vitro* model of the BBB, and also determined the contribution of different mechanisms of endocytosis to the passage of Aβ_42_, pS8-Aβ_42_ and iso-Aβ_42_ through the endothelium. It was found that pS8-Aβ_42_ and iso-Aβ_42_ are better transported by BBB endothelial cells than Aβ_42_ (Figure 1), which may be one of the factors determining the ability of modified forms of Aβ to influence cerebral amyloidogenesis when administered intravenously (Kozin et al., 2013; Barykin et al., 2018).

The main mechanism of transport of Aβ from the bloodstream to the brain is caveolin-dependent endocytosis (Zhu et al., 2018). Indeed, the inhibitor of this form of endocytosis, filipin, suppressed the transport of Aβ_42_, pS8-Aβ_42_, and iso-Aβ_42_ from the upper to lower compartment to the same extent (Figure 4D). Strikingly, the addition of chlorpromazine, which is an inhibitor of clathrin-dependent endocytosis, significantly suppressed the transport of iso-Aβ_42_ (Figure 5D). Thus, the transport of iso-Aβ_42_ may also be dependent on clathrin endocytosis. Also, the contribution of clathrin endocytosis was found for Aβ_42_ and pS8-Aβ_42_, but less pronounced than for iso-Aβ_42_. The involvement of clathrin-dependent endocytosis in transport of proteins from the bloodstream to the brain was previously shown for transferrin and insulin receptors (Roberts et al., 1992; Goulatis and Shusta, 2017; Ayloo and Gu, 2019; Pemberton et al., 2022), but not for beta-amyloid peptides. There is also evidence that LRP-1 can mediate Aβ transport in both directions (Pflanzner et al., 2011). It is possible that in the bEnd.3 cell line some part of the molecules of this receptor is present on the luminal side, which could explain the slight effect of the inhibitor on the transport of Aβ_42_ and pS8-Aβ_42_.

It is assumed that RAGE plays a major role in the transfer of Aβ from the circulatory system to the brain. It was previously shown that in cells expressing RAGE an inhibitor of this receptor, FPS-ZM1, prevented oxidative stress induced by Aβ_40_ and Aβ_42_ (Deane et al., 2012). However, the effect of FPS-ZM1 on the transport of Aβ and its isoforms across the BBB endothelium *in vitro* has not been studied. We found that FPS-ZM1 reduced the passage of Aβ_42_ through the endothelium of the BBB (Figure 6), which correlates well with data obtained previously for Aβ_42_ *in vivo* (Deane et al., 2003, 2012). FPS-ZM1 also inhibited the transport of pS8-Aβ_42_ and iso-Aβ_42_, but the effect of this inhibitor on the passage of pS8-Aβ_42_ was less pronounced than for other isoforms (Figure 6D). Thus, it appears that RAGE is the major receptor in the transport of both Aβ and its modified forms across the BBB.

Since Aβ_42_, pS8-Aβ_42_, and iso-Aβ_42_ differed in their ability to penetrate the cell monolayer, we decided to compare the ability of these isoforms to interact with RAGE. There is relatively little data in the literature on the interaction parameters of Aβ with RAGE. Thus, in cell cultures, the dissociation constants of RAGE with Aβ_40_ and Aβ_42_ were 75 ± 5 nM (Deane et al., 2012) and 92 ± 40 nM (Chellappa et al., 2021), respectively. For purified RAGE, a dissociation constant with Aβ_40_ was shown to be 57 ± 14 nM (Yan et al., 1998). Using the surface plasmon resonance method, it was revealed that sRAGE binds Aβ_42_ oligomers with a Kd of 17 nM (Chen et al., 2007), and the Kd for endogenous soluble RAGE (esRAGE) and Aβ_42_ was 44.9 nM (Sugihara et al., 2012). Thus, direct measurements of the interaction of Aβ_42_ monomers and its isoforms with RAGE have not been previously carried out. The interaction constants obtained for Aβ_42_ are an order of magnitude higher compared to constants estimated in other systems. This may be due to the fact that in our experiments stabilizing agents and other additives that are far from physiological were used, which could affect the obtained constants. Nevertheless, this model allowed us to compare the binding of different isoforms with RAGE in the same conditions. Across the three Aβ_42_ isoforms, we found that RAGE demonstrates the highest affinity to Aβ_42_ and the lowest to iso-Aβ_42_ (Figure 7). These data are in good agreement with the results of computer modeling that we obtained earlier, according to which sRAGE has the lowest calculated Kd value with Aβ_42_ and the highest with iso-Aβ_42_ (Tolstova et al., 2022). The obtained Kd values correlate with the accumulation of amyloid peptides inside cells (Figure 9). We also found that RAGE interacts with Aβ_17 − 42_, but not with Aβ_1 − 16_, and the binding constant of the receptor with Aβ_17 − 42_ was an order of magnitude smaller than the binding constant with the full-length Aβ_42_ peptide. Previously, we observed a similar pattern in the interaction of Aβ with Na^+^/K^+^-ATPase: binding to the enzyme was detected for Aβ_17 − 42_, but not for Aβ_1 − 16_ (Barykin et al., 2018). Probably, the hydrophobic C-terminal fragment Aβ_17 − 42_ makes a major contribution to the binding of Aβ_42_ to various protein molecules, while Aβ_1 − 16_ modulates this interaction.

Apparently, the high affinity of Aβ_42_ for RAGE is the reason for its accumulation in cells and lower transport efficiency compared to other isoforms, while the isoforms with lower affinity for the receptor are more easily transported across the endothelial cell and are able to dissociate from the receptor on the abluminal side. This mechanism was previously shown for the passage of antibodies to the transferrin receptor across the BBB (Yu et al., 2011; Goulatis and Shusta, 2017). High-affinity antibodies against the transferrin receptor cause the antibody-receptor complex to be mainly directed to lysosomes, and those that undergo transcytosis remain associated with the receptor on the abluminal side. Low-affinity antibodies undergo transcytosis and dissociate on the abluminal side to a greater extent (Yu et al., 2011; Goulatis and Shusta, 2017). Similar studies focusing on drug delivery to the brain showed that transferrin-containing nanoparticles with high avidity for the transferrin receptor remained tightly associated with endothelial cells, whereas low avidity nanoparticles dissociated from the receptor after transcytosis (Wiley et al., 2013). In bEnd.3 cells, it was shown that strong binding of ligands to LRP-1 triggers internalization leading to endo-lysosomal sorting and degradation of ligand-receptor complex, while ligands with moderate binding strength to the receptor were transported across the endothelium (Tian et al., 2020). Thus, the stronger binding of Aβ_42_ to RAGE may be the reason for its lowest transport efficiency of all isoforms across the bEnd.3 cell monolayer. Another factor influencing Aβ transport across the BBB may be different degrees of enzymatic degradation of Aβ isoforms. Thus, isomerization of the aspartate residue in Aβ has been shown to prevent its proteolysis in lysosomes (Lambeth et al., 2019). PS8-Aβ is resistant to degradation by insulin degrading enzyme, unlike unmodified Aβ (Kummer and Heneka, 2014). We found different affinities of Aβ isoforms for RAGE, which may affect enzymatic degradation.

Once Aβ enters the brain, it exerts multiple effects on its neuronal and glial targets (Mroczko et al., 2018). Phosphorylated and isomerized isoforms of Aβ act differently; as such, iso-Aβ is likely more toxic to cholinergic neurons bearing certain receptor types, such as alpha7 nicotinic acetylcholine receptor (Barykin et al., 2019), and pS8-Aβ is less prone to inhibit Na,K-ATPase (Barykin et al., 2018). Together with different transport rates of these isoforms, a complex interaction emerges. Aβ_42_ has also been shown to bind to pyramidal neurons after administration into the blood (Clifford et al., 2007). However, the distribution of blood-derived Aβ isoforms in the brain is a subject for future research.

Aging may cause the appearance of modified Aβ isoforms (Moro et al., 2018). Thus, with age, isomerized and deaminated proteins accumulate, and the balance of phosphorylation/dephosphorylation is disrupted (Barykin et al., 2017). Pathogenic forms of Aβ carrying post-translational modifications can arise in the blood and then penetrate the brain, induce aggregation of endogenous beta-amyloid and cause AD pathology. Thus, the appearance of modified forms may precede the formation of plaques and occur in the early stages of the disease. We hypothesize that PTMs are more relevant to sporadic Alzheimer's disease than to familial Alzheimer's disease. However, genetic mutations can also lead to disruption of the PTM process if these mutations affect Aβ-modifying enzymes.

According to our data, phosphorylated and isomerized Aβ are transported more efficiently across the endothelium of the BBB than the unmodified peptide. The RAGE receptor was found to be essential for the transport of both Aβ_42_ and its isoforms across the BBB. Differences in the transport of Aβ_42_, pS8-Aβ_42_, and iso-Aβ_42_ may be due to different mechanisms of endocytosis or different affinities of these isoforms for the RAGE receptor. The mechanisms of transport of Aβ_42_, pS8-Aβ_42_ and iso-Aβ_42_ across the BBB should be taken into account when developing agents for the treatment of AD. Thus, the data obtained may contribute to understanding the causes of the disease, as well as to the search for new drugs that prevent the accumulation of pathogenic Aβ isoforms in the brain.

Limitations: The use of higher than physiological concentrations of Aβ (1 μM) to study its transport is a limitation of this article. However, no saturation transport occurs at 1 μM (Supplementary Figure 1), which justifies the use of this concentration in our study. There are also a number of studies that use high concentrations of Aβ to study its transport across the BBB (Shackleton et al., 2016; Dal Magro et al., 2019; Shubbar and Penny, 2020; Zinchenko et al., 2020). Last, our study is the first to measure all isoforms of Aβ using single ELISA, and the sensitivity of our assay does not allow to measure lower concentrations.

## Data availability statement

The original contributions presented in the study are included in the article/Supplementary material, further inquiries can be directed to the corresponding author.

## Author contributions

KV: Formal analysis, Investigation, Methodology, Writing – original draft. IP: Conceptualization, Investigation, Methodology, Supervision, Writing – review & editing. VM: Conceptualization, Funding acquisition, Project administration, Resources, Supervision, Validation, Writing – review & editing. EB: Conceptualization, Formal analysis, Investigation, Methodology, Writing – original draft. AM: Conceptualization, Funding acquisition, Project administration, Resources, Supervision, Writing – review & editing.
