# Supplementary material for: Post-translational modifications of beta-amyloid alter its transport in the blood-brain barrier in vitro model
Source: Front Mol Neurosci. 2024 Mar 7;17:1362581. doi: 10.3389/fnmol.2024.1362581 (PMC10954796; doi:10.3389/fnmol.2024.1362581)
Supplement: Supplementary file 1 [file Data_Sheet_1.PDF]

Supplementary Material

Post-translational modifications of beta-amyloid alter its transport across the blood-brain barrier

Kseniya B. Varshavskaya, Irina Yu. Petrushanko, Vladimir A. Mitkevich*, Evgeny P. Barykin and Alexander A. Makarov

*** Correspondence:** Vladimir A. Mitkevich: mitkevich@gmail.com

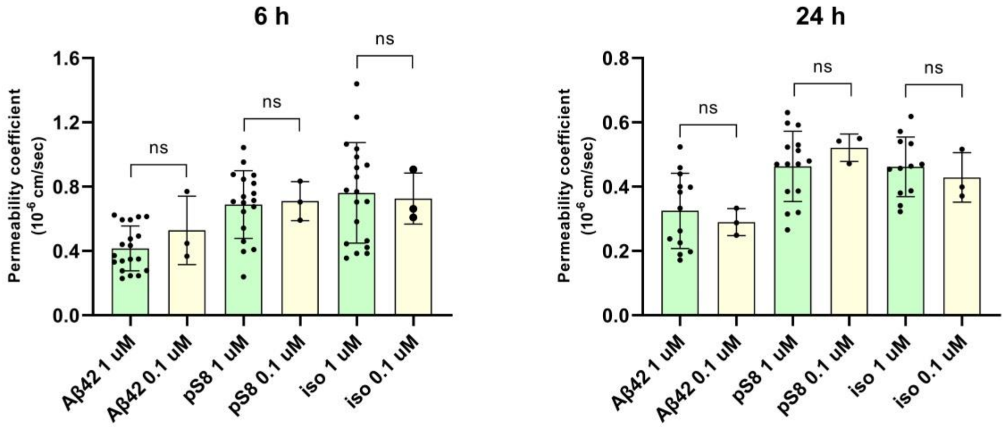

Supplementary Figure 1. Permeability coefficient of amyloid beta isoforms after incubation with 1 µM or 0.1 µM for 6 and 24 hours. Ns - not significant.

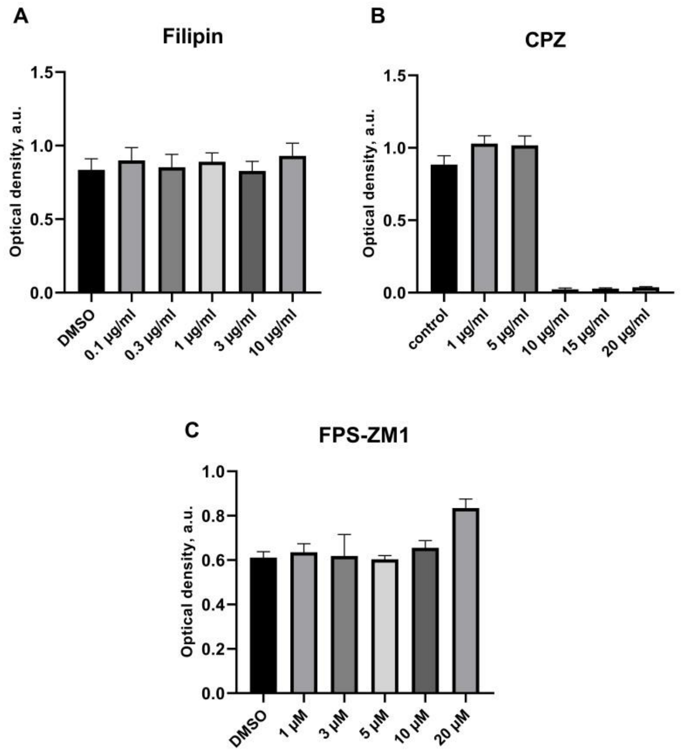

Supplementary Figure 2. Testing the toxicity of filipin **(A)**, chlorpromazine **(B)** and FPS-ZM1 **(C)** for bEnd.3 cells. Cells were incubated with various concentrations of inhibitors or an equivalent amount of DMSO for 24 hours. Toxicity was assessed using MTT (Filipin) and WST (FPS-ZM1 and chlorpromazine) tests. Optical density at various inhibitor concentrations is presented. Number of replicates in each group n = 3-4.

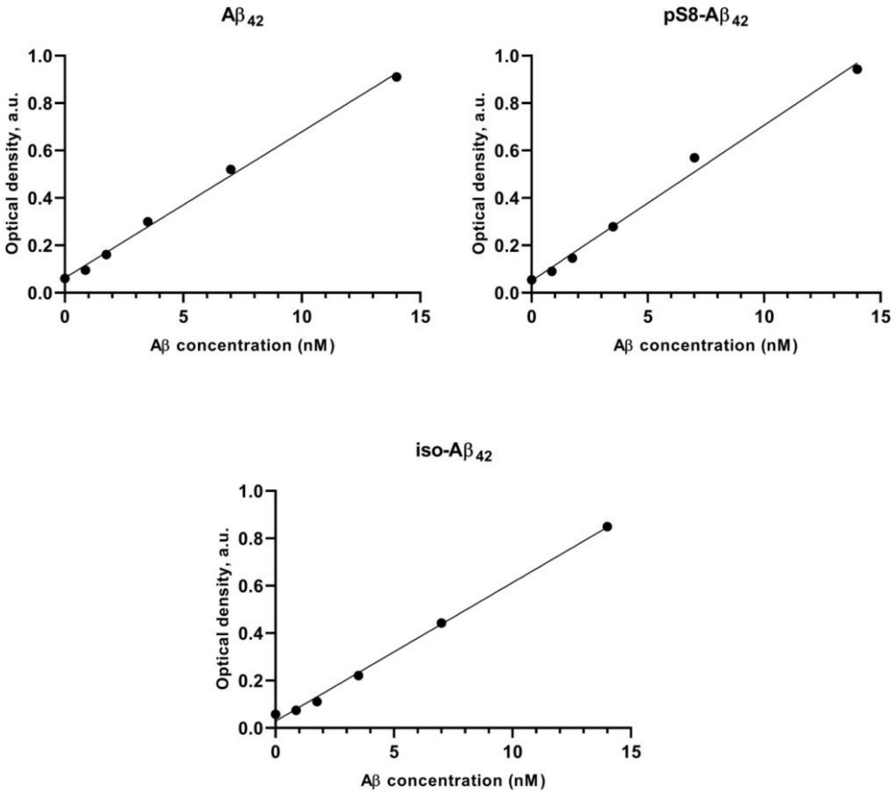

Supplementary Figure 3. ELISA calibration curves for $A\beta_{42}$, pS8-$A\beta_{42}$ and iso-$A\beta_{42}$.
